# Magnetic frustration of graphite oxide

**DOI:** 10.1038/srep44690

**Published:** 2017-03-22

**Authors:** Dongwook Lee, Jiwon Seo

**Affiliations:** 1 Department of Physics, University of Cambridge, J.J. Thomson Avenue, Cambridge CB3 0HE, United Kingdom; 2 Physics and Applied Physics, School of Physical & Mathematical Sciences, Nanyang Technological University, 21 Nanyang Link, 637371, Singapore; 3 Department of Physics and Applied Physics, Yonsei University, Seoul, 120-749, Korea

## Abstract

Delocalized *π* electrons in aromatic ring structures generally induce diamagnetism. In graphite oxide, however, *π* electrons develop ferromagnetism due to the unique structure of the material. The *π* electrons are only mobile in the graphitic regions of graphite oxide, which are dispersed and surrounded by *sp*^3^-hybridized carbon atoms. The spin-glass behavior of graphite oxide is corroborated by the frequency dependence of its AC susceptibility. The magnetic susceptibility data exhibit a negative Curie temperature, field irreversibility, and slow relaxation. The overall results indicate that magnetic moments in graphite oxide slowly interact and develop magnetic frustration.

Carbon, one of the most abundant elements on earth, forms millions of chemical compounds. Among them, graphene-based carbon compounds such as graphite have been used in many industries, and they still hold the attention of physical and chemical engineers owing to their outstanding properties such as high specific surface area, lubricating ability, sorption, catalytic characteristics, and deaccelerating effects^1^,^2^,^3^. Aromatic rings in graphitic compounds, which are units of the graphitic structure, are considered diamagnetic units because a current along the ring produces diamagnetism when an external magnetic field is applied. Thus, graphitic materials generally exhibit diamagnetic properties. However, recent reports on the various ferromagnetic properties of graphitic compounds have sparked research on carbon magnetism. Their origins are as follows: proton-irradiated graphite^4^,^5^, graphite with zigzag edges^6^,^7^,^8^, defect-induced graphite^9^,^10^, hydrogen-mediated graphite^11^,^12^, and others^13^,^14^,^15^,^16^. These materials are conductors and have layered structures like graphite. Thus, the magnetic properties of ferromagnetic carbon systems have been considered together with their electrical properties. The emergence of nano-graphite has resulted in increased interest in nano-graphitic structures^17^,^18^,^19^,^20^,^21^. In nano-carbon structures, the electrical conductivities are less important. Rather, the edge condition and functional groups are more received^22^,^23^,^24^. However, no thorough research exists on the mixed structure of nano-graphite and non-graphitic compounds. Graphite oxide (GO)^25^,^26^,^27^ has attracted huge interest owing to its structural properties, which induce various properties such as optical, thermal, and dielectric properties, as well as mass production of graphene by reduction^28^,^29^,^30^,^31^. The graphitic regions in GO are dispersed among non-graphitic carbon atoms. The co-existence of graphitic and non-graphitic regions in GO results in an abundant surface area; hence, GO has been studied in the energy-storage industry and for application in sensors^32^,^33^,^34^,^35^.

The ferromagnetism in GO has been carefully investigated^36^,^37^,^38^,^39^,^40^. In our previous letter^36^, we showed that GO exhibits ferromagnetic behavior owing to the graphitic regions circulated by epoxy groups, and it has an S-shaped M-H curve. Here, we demonstrate the frequency dependence behavior of the AC susceptibility of GO and extract spin-glass parameters through scaling analysis. Its magnetic moment slowly changes with time at low temperature and GO illustrates glass-like behavior at these temperatures. This behavior is caused by interactions between the graphitic regions in GO.

To characterize materials using solid-state NMR, it would be better to compare the high-power decoupling (HPDEC) spectrum of samples with their cross-polarization magic angle spinning (CP/MAS) spectrum. In the HPDEC mode, the coupling between carbon and hydrogen atoms is broken, while in CP/MAS, the signal of carbon atoms is enhanced by the cross-polarization of carbon and hydrogen atoms. Thus, by comparing HPDEC data with CP/MAS data, we can determine which peaks come from carbon atoms near hydrogen atoms^26^,^41^.

Figure 1 shows the experimental ^13^C MAS spectrum of GO acquired with HPDEC and CP/MAS with a 3550 *μ*s contact time, during which the spectrum has a maximum value. There are peaks at roughly 60, 70, 120, 130, and 190 ppm. The intensity of the peak at 70.04 ppm is increased in the CP/MAS mode, while the peak intensity at 58.92 ppm does not differ between the HPDEC and CP/MAS modes^26^,^41^,^42^. The two small peaks at 187.94 and 193.25 ppm demonstrate similar behavior. The peak at 193.25 ppm does not change with the modes. However, the peak at 187.94 ppm increased in the CP/MAS mode. Therefore, we conclude that the peak at 58.92 ppm is due to the epoxy groups, the peak at 70.04 ppm is due to the hydroxyl groups, the peak at 130 ppm is due to the stable double bonds^41^, the peak at 193.25 ppm is due to the ketone groups^26^,^43^, and the peak at 187.94 ppm might be a result of the chemical groups containing carbon and hydrogen atoms such as -COOH. The broad peak around 130 ppm is due to graphitic rings. The NMR data indicate that GO is composed of graphitic and non-graphitic regions.

Figure 2(a) compares the Raman spectra of GO and H-GO. GO exhibits two high peaks at 1440 (D) and 1660 (G) *cm*^−1^. It also exhibits characteristic peaks at 2750 (2D), 2930 (D + G), and 3150 (2G) *cm*^−1^. After thermal treatment, the characteristic peaks of GO at the above positions were diminished. The diminished 2D peak indicates that the graphitic regions in GO were damaged. In addition, the D peak at 1440 *cm*^−1^ increased and grew broader, implying that more defects were produced during the heat treatment.

Figure 2(b) illustrates the C K-edge X-ray absorption near-edge structure (XANES) spectra of GO, H-GO, and polycrystalline graphite acquired in the total electron yield (TEY) mode at 298 K. The spectrum of graphite has two main peaks; the first peak at 285 eV is due to the π* state of C=C^44^,^45^,^46^ and the second at 292 eV is assigned to C=C σ* antibonding^44^,^45^,^46^. The spectrum also includes the peak from the core-exciton, a sharp peak at 291.6 eV. The spectrum of GO is different from that of graphite; peak A is due to unoccupied π* states^44^, peak B at 287 eV results from aliphatic C-OH^47^, peak C at 289 eV is due to C-O-C bonding, and peak D at 291.8 eV indicates unoccupied σ* states^44^. The peaks in the H-GO spectrum of H-GO differ from those in the other samples. Here, peak A (the π* state of C=C) is suppressed because the graphitic structure in GO was damaged during the heat treatment, which is consistent with the Raman results in Fig. 2(a). The height of peak B’ at 288 eV near peak B increases due to the generation of aliphatic C-H such as cyclohexane^47^. Peak C is nearly eliminated because epoxy groups decomposed during the thermal treatment and peak D shifts to 293 eV.

DC magnetic susceptibility measurements were performed using a superconducting quantum interference device (SQUID) magnetometer in zero-field-cooled (ZFC) and field-cooled (FC) modes with various applied magnetic fields. Figure 3(a) shows the susceptibilities of GO as a function of temperature. The susceptibility curves in ZFC mode exhibit a characteristic maximum and a thermal hysteresis (multiple paths) below it. As the temperature decreases, the FC curves become saturated. As the field increases from 100 to 800 Oe, the peaks become broader and shift from 25 to 15 K. This feature often appears in relaxors such as spin-glass materials^48^,^49^,^50^,^51^,^52^,^53^. The inset in Fig. 3(a) depicts the curves across a wide temperature range. The curves were fitted by the Curie-Weiss law, and the Curie temperatures *θ*_*c*_ at different magnetic fields are given in Table 1. A negative *θ*_*c*_ indicates the presence of antiferromagnetic interactions. H-GO does not show thermal hysteresis in Fig. 3(b). Instead, it exhibits a paramagnetic behavior. The Curie temperatures of H-GO are 0.56 and 0.51 K at 250 and 750 Oe, respectively.

In H-GO, the Bohr magneton number *n*_*B*_ is below the order of 10^−4^, i.e., negligible compared to that of GO. In addition, H-GO exhibits a linear curve in the M-H measurements in Fig. 3(c), unlike GO, which has an S-shaped curve. The above data indicate that there are ferromagnetic as well as antiferromagnetic interactions in GO and the magnetic properties of GO were destroyed during the heat treatment.

Further investigation of the magnetic behavior of GO was carried out using the Almeida-Thouless (AT) law and critical scaling analysis. The irreversibility temperatures (*T*_*ir*_) identified by the splitting of the ZFC and FC curves in Fig. 3(a) are plotted as a function of the applied field in Fig. 4(a), and they follow the AT line (H^2/3^) (Fig. 4(a) (inset))^54^. By extrapolating the AT line to H = 0, we determine a freezing temperature *T*_*f*_ = 24.5 ± 0.05 K. The order parameters of the spin glass were obtained using critical scaling analysis^55^. Normalized q(T) values, i.e., the deviation of the observed susceptibility from paramagnetic behavior, were extracted from both FC and ZFC data and plotted at various fields, as shown in Fig. 4(b). The FC data was fitted to q = |*t*|^*β*^ as t → 0, yielding q = H^2/(1+*γ*/*β*)^ near *T*_*f*_^52^ with *β* = 0.5 ± 0.1 and *γ* = 9 ± 1. These parameters vary from those associated with a spin-glass in that *β* = 0.2 ± 0.1 and *γ* = 4.5 ± 0.5 in 2D spin-glass and *β* = 0.5 ± 0.2 and *γ* = 4.0 ± 0.4 in 3D spin-glass^56^. This variation implies that GO may exhibit both 2D and 3D spin-glass behavior, i.e., both 2D and 3D glass behaviors are present in GO.

Figure 4(c) shows the in-phase component *χ*’(T, *ω*) of the AC susceptibility of GO between 15 and 35 K in the frequency range 10 ≤ *ω* ≤ 1000 Hz. The measurements were taken in zero field cooling (ZFC) conditions with a 10 Oe AC field at different frequencies. The *χ*’(T, *ω*) curve exhibits a characteristic pronounced maximum with amplitude and position, depending on the frequency. As *ω* increases, the *χ*’ exhibits a maximum with amplitude and position, which is similar to the AC susceptibility behavior of glassy systems^57^,^58^,^59^. The AC susceptibility’s dependence on the frequency is a sign of slowing in the magnetization dynamics. The maximum of the peak shifts towards higher temperatures with increasing frequencies, which is a common feature for spin glasses^57^,^58^,^60^. The inset shows that the freezing temperature depends on the frequency. However, H-GO does not show any characteristic peak in the AC susceptibility curve (Fig. 4(d)).

To determine whether the GO sample has a slow relaxation, its FC relaxation effects were examined. First, a magnetic field of 100 Oe was applied to the samples at room temperature and then cooled to 7 K. The measurements were performed just after the magnetic field was removed. In Fig. 4(e), the magnetization of GO decreases exponentially with time, whereas H-GO magnetization exhibits no time dependence. The magnetization curve of GO was fitted as 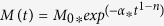, where n ~ 0.56 is similar to that of a dilute spin system^52^. Unlike GO, H-GO does not show slow relaxation.

In the previous letter, we demonstrated that the spin densities are mainly localized in the graphitic domains in GO, as shown in Fig. 4(f). When the distance between the graphitic domains becomes short enough, the spins in the domains can be coupled ferromagnetically and/or antiferromagnetically. According to our EDX analysis results, the C:O ratio of the sample is 6:2.3 and the ratio of *sp*^2^-carbon to *sp*^3^-carbon is 1.4. The graphitic domains are small because the oxygen coverage is so high. Thus, the interaction among the graphitic domains may be ferromagnetic and/or antiferromagnetic. However, the graphitic and non-graphitic regions in GO depends on the preparation method and the degree of oxidation^27^. The magnetic spin densities might be also influenced by the degree of oxidation and the preparation methods, which also determine the area and distribution of graphitic domains. In addition, the graphitic domains, which are hydrophobic, are different from hydrophilic reacted regions in GO. Thus, the amphiphilicity of GO might make the magnetic properties of GO more complex.

GO exhibits field irreversibility and slow relaxation. The DC susceptibility data show that the sample has a negative Curie temperature and exhibits field irreversibility in addition to slow relaxation. The frequency-dependence of AC susceptibility corroborates the spin-glass behavior. The destruction of epoxy groups gets rid of the magnetic properties of GO. Although the origin of this magnetism is not clear, it is apparent that the epoxy group plays a fundamental role in the observed magnetism. The overall results suggest that the magnetic moments in GO slowly interact and exhibit glass-like behavior.

## Method

The GO samples were prepared by the Staudenmaier process^27^. GO was heated at 250 °C for 24 h (H-GO). The structural properties of the sample were characterized using solid-state nuclear magnetic resonance (NMR). The ^13^C spectra were obtained at 9.4 Tesla using a Bruker AVANCE 400 MHz spectrometer and 4 mm zirconia MAS rotors spun in air at 6 kHz. ^13^C MAS spectra with HPDEC were acquired at 100 MHz with 100-kHz, 90 °C pulses with a duration of 1.25 *μ*s. ^1^H-^13^C CP/MAS spectra were recorded at different contact times (50–5050 *μ*s) with increases of 500 *μ*s. The peaks were most intense at 3550 *μ*s. Raman spectra were collected at an excitation wavelength of 514 nm (Renishaw, RM-1000Invia, 2400 l/mm). X-ray absorption near edge structure (XANES) measurements were performed on the BACH beamline at ELLETRA in Italy. The magnetic properties of the samples were characterized with a Quantum Design MPMS 1802 magnetometer and a Lakeshore 7000 Series AC Susceptometer.

## Additional Information

**How to cite this article**: Lee, D. and Seo, J. Magnetic frustration of graphite oxide. *Sci. Rep.* **7**, 44690; doi: 10.1038/srep44690 (2017).

**Publisher's note:** Springer Nature remains neutral with regard to jurisdictional claims in published maps and institutional affiliations.

## Figures and Tables

**Figure 1:**
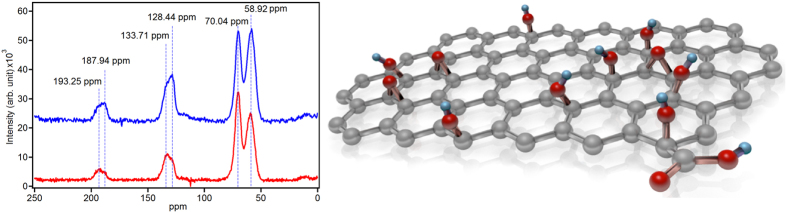
(Left) Solid-state ^13^C NMR spectra of GO. High-power decoupling (red) and CP/MAS using 3550 *μ*s contact time (blue). (Right) Schematic of GO. Red balls represent oxygen atoms and cyanide balls represent hydrogen atoms.

**Figure 2:**
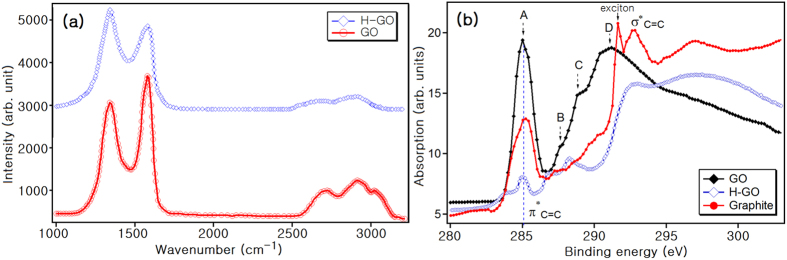
(**a**) Raman spectra of GO and H-GO (**b**) C K-edge XANES spectra of GO, H-GO, and graphite at 298 K.

**Figure 3:**
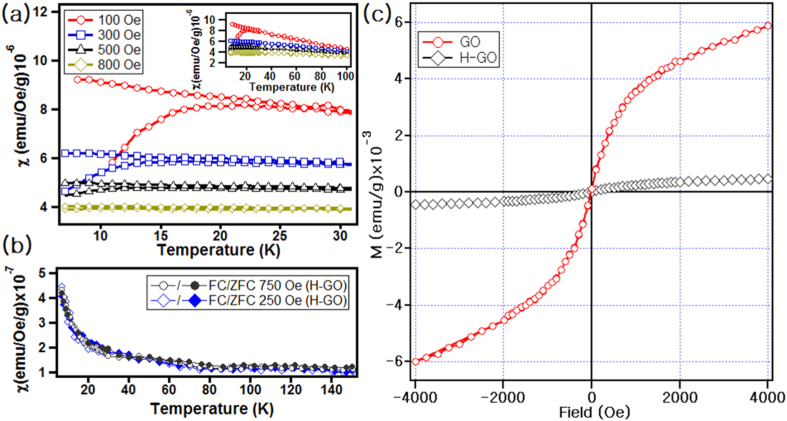
(**a**) Magnetic susceptibility of GO as a function of temperature at various field strengths. The inset shows the susceptibility of GO in the temperature range 6 ≤ T ≤ 180 K. (**b**) Magnetic susceptibilities of H-GO as a function of temperature at various field strengths. (**c**) Magnetization versus magnetic field curves of GO and H-GO.

**Figure 4:**
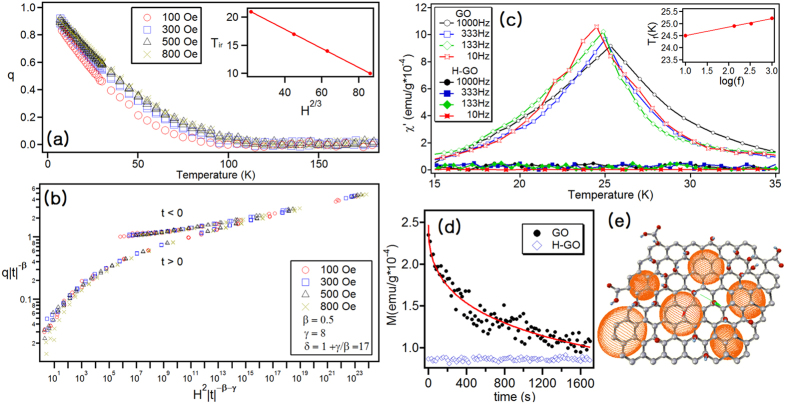
(**a**) Scaling analysis of data in Fig. 3(a). Spin-glass order parameter q as a function of temperature. The inset shows that irreversibility temperatures *T*_*ir*_ is linearly dependent on 1/H^2/3^ and follows the Almeida-Thouless Law (H^2/3^). (**b**) Critical scaling for GO. (**c**) AC susceptibility curves of GO with different AC fields. Frequency ranges from 10 to 1000 Hz. The inset displays a graph of the freezing temperature (*T*_*f*_) vs. frequency. (**d**) AC susceptibility curves of H-GO with different AC fields. (**e**) FC relaxation effect in GO and H-GO at 7 K. (**f**) Schematic for glass-like behavior of GO. Red balls are oxygen atoms and cyanide balls are hydrogen atoms. Orange spheres indicate the magnetic domain developed in the graphitic regions in GO.

**Table 1:** Curie temperature (*θ*_*c*_) and effective Bohr magneton number (*n*_*B*_) of the samples obtained with different magnetic fields.

Sample	GO	GO	GO	GO	H-GO	H-GO
Field (Oe)	100	300	500	800	250	750
*θ*_*c*_ (K)	−52.2	−40.2	−19.5	−12.0	0.56	0.51
*n*_*B*_	1.02 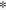 10^−3^	1.23 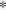 10^−3^	1.35 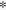 10^−3^	1.32 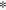 10^−3^	8.34 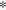 10^−5^	1.43 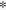 10^−4^

In the Curie-Weiss equation (*χ* = *C*/(*T* − *θ*_*c*_)), C is the Curie constant and is described as follows: C = (*n*_*B*_)^2^(*μ*_*B*_)^2^/3*k*_*B*_, where *μ*_*B*_ is the Bohr magneton and *k*_*B*_ is the Boltzmann constant.
